# Aqua Calcis

**Published:** 1886-05

**Authors:** George J. Friedrichs


### AQUA CALCIS.

BY GEORGE J. FRIEDRICHS, M. D., D. D. S.

READ BEFORE THE NEW ORLEANS ODONTOLOGICAL SOCIETY.

My attention was attracted to the subject of Aqua Calcis by the perusal of an article entitled "Dental Nutrition," which appeared in the November, 1885, edition of the INDEPENDENT PRACTITIONER, and believing that a few remarks upon this subject may not be entirely without interest to this body, I have hastily collated, for your consideration, a few facts and deductions bearing upon the matter involved.

In the above-named paper, the two following postulates were advanced:

First. "That the solution of lime faithfully administered—simple, plain lime-water—will be so decomposed and reorganized within the system as to furnish the elements necessary to harden and reconstruct poor, soft, chalky teeth."

And secondly. "That the general diet of the residents of New Orleans, with cistern water used exclusively for potable purposes, constituting an environment differing considerably from that of any other portion of the country," results "in a greater amount and degree of softened, decalcified teeth than are to be found elsewhere."

Now, in order that these assertions might have carried with them some plausible evidence of established truth, it is reasonable to presume that they ought to have been accompanied by reliable statistics, or at least fortified by the testimony of others residing in the same locality, to whom this unusual predominance of softened, decalcified teeth must have been equally apparent, and whose opportunities for investigation, both as to causes and effects, must necessarily have been equally as good. The mere dictum and observation of any one individual, no matter how well qualified he may be, cannot be supposed to counterbalance the experience and conclusions of the profession at large, unless his theories are based upon a foundation of incontrovertible evidence. In this case no such evidence has been adduced. On the other hand, after many years of experiment and discussion, quite contrary views to those announced now obtain with the best minds of the profession, and it is pretty generally accepted as an ascertained fact that neither diet nor geographical location has anything whatever to do with the formation of the teeth.

I will now relate a case in my own practice, bearing upon the question of the inferior quality of teeth, alleged to be peculiar to residents of New Orleans and its vicinity, which I present for your consideration, and from which you may draw your own conclusions.

About two years ago I was called upon by one of my patients, bringing her little daughter with her. I saw that there was considerable anxiety felt on the part of the mother in regard to the result of my diagnosis. Upon examination, I found an unusual deposit of salivary calculus, incrusting the lower incisors to such an extent that they were apparently a solid mass of tartar; hence, the mother's anxiety, she being unable to account for what had become of the teeth which were there before. With the aid of a scaling instrument I soon demonstrated, to her delight, the real condition of affairs. The parents of the child have been residents of New Orleans for years. The child herself was born here, has subsisted solely upon city fare, and has used nothing but cistern water for potable purposes. The question here presents itself: Where did this unusual deposit of lime, which was found incrusted upon her teeth, come from? I admit that this case, as to the amount of tartar deposited, is an isolated one, and the first met with in my practice; but the deposit of tartar in a minor degree, requiring removal in order to prevent absorption and recession of the gums, is not an uncommon occurrence amongst the population here, a fact well known to every dentist practicing in the City of New Orleans, and which appears to controvert the assumption that the inhabitants of this section, in consequence of their peculiar environment, relying solely upon cistern water for drinking purposes, are peculiarly liable to a greater amount and degree of softened, decalcified teeth, than are to be found elsewhere.

I will now discuss the general theory upon which this proposition is advanced; *i. e.*, the utility of administering simple, plain limewater in order "to harden and reconstruct poor, soft, chalky teeth." I will commence by quoting the opinion of Dr. Magitot, who asserts "that the nature of drinks used by populations play, as we have before hinted, but a secondary role, or is perhaps nil." And in the words of Prof. Truman: "The fact that hens must be occasionally fed on lime in order that shells may be formed, cannot be regarded as adding any strength to the argument in favor of lime assimilation. The shells of eggs cannot be held as organized products, and hence they bear no more relation to the hard tissues of the animal organization than a calcareous deposit in the mouth resembles the tooth upon which it is deposited. That increased density can be effected by the presentation of food containing the necessary elements, there can be no doubt, but that it can ever be accomplished by direct administration of the inorganic, I hold to be impossible. Careful observation, through a series of years, in lime-water districts, leads to the conclusion that assimilation cannot be carried on in this way, and that it is waste of energy to do so."

Dr. W. C. Barrett, in a paper read on this subject before the American Dental Association, says: "Corroborative evidence, when looked for with biased judgment, was not lacking, and dentists had tales to relate of the most stupendous changes effected in the development of children through a judicious administration of the lacto-phosphate of lime to the mother during pregnancy. The instances in which such results seemed apparent were cited, while those in which no effect resulted were not included in the category, or were attributed to a want of faithfulness in taking the prescription. We believe that which we desire to believe, and there is no difficulty in finding apparent confirmatory evidence to sustain the most absurd postulates when one sets out with the determination to do so."

And again: "Any man, or pregnant woman, who lives upon almost any diet that is sufficient to sustain life, will, if the nutritive organs be in proper condition, find more than is necessary of these elements (*i. e.*, lime-salts) to keep the osseous system in good condition." After giving some of the physiological reasons why the administration of the earthy phosphates for nutritive purposes must be a mistake, he concludes by stating: "If they act at all, it must be remedially, and if they are used they should be intelligently prescribed, like any other agent, and only for their medicinal properties. I have no knowledge that they have any very decided medicinal virtues, and, therefore, can see no excuse for dispensing such inert compounds."

Prof. Morgan, of the Vanderbilt University, Nashville, in the discussion of the subject before the Southern Dental Association, expressed his convictions thus: "A great deal has been said in the last few years, and some allusion has been made to proper diet in general, and to diet having the proper amount of bone-making material, and the impression has been received by the dental profession in this country everywhere that there is too small an amount of bone-making material in all food, and in all bread particularly. I accepted the doctrine when first promulgated, but after examination I came to the conclusion that we are entirely wrong on that subject. Take rice, which has $\frac{91}{100}$ of one per cent. of saline matter in it, and if you sit down and make a simple calculation, there is more bone-making material in rice, if a man were to consume a quarter of a pound a day, than is necessary to build up and make the best osseous structure that a man ever had."

According to the average dietaries for adults in full health, collected by Dr. Playfair (*London Chemical News*, May 12th, 1865), about twenty grammes of mineral matter are daily introduced with the food; and so I might go on enumerating testimony in support of these now universally accepted facts, sufficient to fill a volume.

Lime-water is a perfectly clear and transparent liquid, inodorous, and has an astringent and somewhat alkaline taste. It changes vegetable blues to green. It differs from the solutions of the alkalies in being destitute of caustic properties. On the other hand, it has a powerful astringent and styptic effect; hence, even in overdoses, it never disorganizes the animal tissues. Its astringency is not shown by a contraction of the parts to which it is applied; it appears rather to control their secretive action, rendering them dry and pale. When absorbed it has a similar effect upon the glandular organs, diminishing their secretions in a remarkable degree.

Therapeutically, it has been prescribed for a variety of ailments. In 1739 the British Parliament decreed a national recompense of £5,000 sterling to Mrs. Johanna Stephens for her lithontriptic remedy, which consisted of egg-shells, soap, and several aromatic bitters. This preparation, however, was found so nauseous and so distressing to the stomach that various substitutes were proposed for it, among which the most simple was lime-water. The writings of Gaitshel, Blanc, Whytt and Alton may be cited in favor of the efficacy of this liquid in calcareous affections. Here we see lime-water administered for the purpose of dissolving calcareous accretions in the bladder, yet, on the other side, we are told, when properly administered, it will induce a recalcification of the teeth. Most wonderful remedy!

Nutrition is a tissue-function; the act of this process is the appropriation from without of the materials which enter into the composition of the living frame, or of others which may be converted into them in the interior of the body. The difference between the protoplasmic elements that originate a tooth and those which originate bone, we may never be able to distinguish; yet we know there is a force that directs to formation as certain as intercepted light will cast a shadow. Over this process or affinity we have no control. The tissues may be bathed or saturated with the lime-salts, but if they be not taken up or appropriated their presence is of no value. You may lead an ox to water, but you cannot make him drink.

I know of no remedy that is more efficacious in neutralizing the acidity of the fluids of the mouth. It is an excellent astringent in flaccidity and sponginess of the gums, preventing putrefaction, and by its mild stimulation inducing a healthy reaction of the general tissues of the oral cavity, in this manner removing the external causes of caries, and thus preventing the progress of decay of the teeth.

If the administration of lime-water is efficacious in hardening decalcified teeth, it ought to be a specific in rachitis; yet we find that its administration in combination with milk is only occasionally beneficial.

If the theory advanced that, on account of the geological formation that environs New Orleans, the inhabitants thereof are deprived of a sufficiency of lime-salts, because they are confined to cistern water for potable purposes which contains no lime, rachitis ought to be more prevalent here than it is; yet, in my professional experience of thirty-six years, but one case of rachitis has come under my observation.

Softened and poorly organized teeth are not confined to any one locality of this country. The best proofs that can be brought forward in evidence of the universal prevalence of poorly organized teeth are the papers published some eight years ago by the advocates of what was then called the "New Departure," who claimed that the large number of softened and decalcified teeth existing throughout the length and breadth of this land—lime-stone districts as well as elsewhere—could not, because of this condition, be saved by means of gold fillings, and averred that plastics must be substituted instead.

In conclusion, I think it can be safely asserted, judging from all the lights we have before us, that the administration of aqua-calcis as an effective agent in supplying the necessary rehabilitating elements has been weighed in the balance andfound wanting; and, as the natural deduction, that New Orleans is more liable, on account of its peculiar environment, to the production of "softened and decalcified" teeth than is the case elsewhere, is a palpable fallacy.

